# Expression of metalloproteinases MMP-2 and MMP-9 is associated to the presence of androgen receptor in epithelial ovarian tumors

**DOI:** 10.1186/s13048-020-00676-x

**Published:** 2020-07-28

**Authors:** Flavia Morales-Vásquez, Rocío Castillo-Sánchez, María J. Gómora, Miguel Ángel Almaraz, Enrique Pedernera, Delia Pérez-Montiel, Elizabeth Rendón, Horacio Noé López-Basave, Edgar Román-Basaure, Sergio Cuevas-Covarrubias, Juan Maldonado-Cubas, Antonio Villa, Carmen Mendez

**Affiliations:** 1 Instituto Nacional de Cancerología, Secretaría de Salud de México, Ciudad de México, Mexico; 2 Departamento de Embriología y Genética, Facultad de Medicina, Universidad Nacional Autónoma de México, Circuito interior, Ciudad Universitaria, Av. Universidad. 3000, C.P. 04510 Ciudad de México, Mexico; 3 Posgrado de la Facultad de Ciencias Químicas, Universidad La Salle, Ciudad de México, Mexico; 4 Hospital Militar de Especialidades de la Mujer y Neonatología. Secretaría de la Defensa Nacional, Ciudad de México, Mexico; 5 Hospital General de México, Ciudad de México, Mexico; 6 División de Investigación, Facultad de Medicina, Universidad Nacional Autónoma de México, Ciudad de México, Mexico

**Keywords:** Ovarian tumors, Metalloproteinase MMP-2, Metalloproteinase MMP-9, Androgen receptor, Estrogen receptor alpha, Progesterone receptor

## Abstract

**Background:**

The current study evaluated the metalloproteinases MMP-2 and MMP-9 expression in epithelial cells and the surrounding stroma in ovarian tumors and the association of MMPs with the histological subtypes, the clinical stage and the presence of steroid hormone receptors. Tumor samples were obtained from 88 patients undergoing surgical cytoreduction of primary ovarian tumors in Instituto Nacional de Cancerología, from México City. The formalin fixed and paraffin embedded samples were processed in order to demonstrate the presence of androgen receptor,estrogen receptor alpha, progesterone receptor, MMP-2,MMP-9 and collagen IV by immunohistochemistry and/or immunofluorescence.

**Results:**

MMP-2 and MMP-9 were differentially expressed in the epithelium and the stroma of ovarian tumors associated to histological subtype, clinical stage and sexual steroid hormone receptor expression. Based on Cox proportional hazard regression model we demonstrated that MMP-2 located in the epithelium and the stroma are independent prognostic biomarkers for overall survival in epithelial ovarian tumors. Kaplan Meir analysis of the combination of AR (+) with MMP-2 (+) in epithelium and AR (+) with MMP-2 (−) in stroma displayed a significant reduction of survival.

**Conclusions:**

The presence of MMP-2 in the stroma of the tumor was a protective factor while the presence of MMP-2 in the epithelium indicated an adverse prognosis. The presence of AR associated with MMP-2 in the tumor cells was a risk factor for overall survival in epithelial ovarian cancer.

## Introduction

Ovarian cancer is the third in frequency and the first in lethality out of all gynecological malignancies and every year about 185.000 women die of this causeworldwide [1]. Its lethality associated with late diagnosis at advanced stages of the disease [2]. Epithelial ovarian cancer comprises almost 95% of ovarian malignancies and according to the histopathology features of the tumor, high grade serous carcinoma (HGSC) is its most frequent presentation, followed by endometrioid, mucinous, low grade serous carcinoma (LGSC) and clear cells subtypes [3]. Risk factors related to this disease are increased age, replacement hormone therapy during menopause and nulliparity. In contrast, the use of oral contraceptives and parity confers protective effects against it [4].

The matrix metalloproteinases (MMPs) are zinc-dependent endopeptidases involved in tissue remodeling in physiological and pathological processes [5]. Particularly, MMP-2 and MMP-9,also known as “gelatinases”, destroy the barriers enclosing a tumor, including collagen IV of the basement membrane and the extracellular matrix, allowing invasion into the surrounding tissue [6, 7]. The activation of zymogen forms of MMPs is mediated by a mechanism that has not yet been fully elucidated. The cleavage by other MMPs anchored to cell membranes and activation by the plasmin cascade has been previously described [8, 9]. The MMPs’ expression is regulated by many factors, including extracellular matrix proteins, growth factors, and cytokines as well as hormonal receptors [10–12]. Previous studies have described the expression of MMPs in epithelial ovarian cancer and evaluated the presence of MMP-2 and MMP-9 in the tumor as potential biomarkers of aggressiveness of the malignancy, registering the disease-free period and the overall survival of patients [13–18].

On the other hand, sexual steroid hormone receptors (androgen receptor -AR-, estrogen receptor alpha -ERα-, and progesterone receptor -PR-) are present in the nucleus of epithelial cells in ovarian carcinoma [19, 20] and their presence has been associated with disease-free and overall survival of the patients [21, 22] with their effect on tumor cell survival having been evaluated in primary cultures of epithelial ovarian tumors [23].

There is evidence of the association of steroid hormone receptors with MMPs expression in breast and prostate cancer [24, 25]. To the best of our knowledge, the association of MMPs expression with the presence of steroid hormone receptor is still controversial in epithelial ovarian cancer. The aim of the current study was to evaluate the frequency of MMP-2 and MMP-9 expression in epithelial tumor cells and the surrounding stroma in ovarian tumors. Additionally, the association of MMPs with the histological subtypes, the clinical stage of the disease according to the International Federation of Gynecology and Obstetrics (FIGO), and the presence of steroid hormone receptors were analyzed. Moreover, the expression of MMP-2 and MMP-9 was evaluated as independent biomarkers for overall survival in patients of the present cohort.

## Material and methods

### Patients and tissue samples

Tissue samples were obtained from 88 patients undergoing surgical cytoreduction of ovarian tumors before any chemotherapy treatment in Instituto Nacional de Cancerologia, from México City. The protocol of the study was approved by the Ethical Board of the Instituto Nacional de Cancerologia 019/060/OMI and Facultad de Medicina, Universidad Nacional Autónoma de México FM/DI/108/2015. The patients who took part in the study were mainly residents of the central region of Mexico and their clinical characteristics were obtained from the hospital clinical records. Their data is summarized in Table 1.

**Table 1:** Clinical characteristic of patients with histological subtypes of ovarian tumors

	SBT	HGSC	Endometrioid	Mucinous	LGSC
Median age (years)	39	56	48	54	54
Menopause	20%	76.1%	52.3%	81.8%	55.5%
FIGO stages					
I	13/20	–	11/21	9/11	4/9
II	1/20	2/21	2/21	–	1/9
III	5/20	14/21	6/21	–	3/9
IV	1/20	4/21	1/21	1/11	1/9
Histological grade					
G1	–	–	3/21	–	–
G2	–	–	14/21	–	–
G3	–	–	2/21	–	–
Surgicaldebulking					
Optimum	20	11	14	10	7
Suboptimum	0	7	6	0	0

*HGSC* high grade serous carcinoma, *SBT* serous borderline tumor, *LGSC* low grade serous carcinoma

In this retrospective study, the formalin fixed and paraffin embedded samples were obtained from the Pathology Department during 2009–2017 and processed in order to demonstrate the presence of, AR,ERα, PR, MMP-2,MMP-9 and collagen IV by immunohistochemistry and/or immunofluorescence. The diagnosis of histological subtypes of epithelial ovarian tumors were obtained from the pathology report present in the patient file. Patients had been diagnosed with primary epithelial ovarian tumors and classified based on previous studies [3] as serous borderline tumors (SBT), high grade serous carcinoma (HGSC), low grade serous carcinoma (LGSC), endometrioid carcinoma and mucinous ovarian carcinoma. Clear cell ovarian carcinomas were not included in the study due to the low number (four) of samples. Overall survival rates were analyzed at the end of 2018.

### Immunohistochemistry and immunofluorescence

Immunostaining techniques were performed on sections from tissue microarrays containing a representative sample of each tumor (4 mm core) as.

previously described [21]. The following primary antibodies were used: anti-AR antibody (Cat. No sc816, Santa Cruz Biotechnology, Santa Cruz, CA), anti-ER alpha antibody (Cat. No sc543, Santa Cruz Biotechnology, Santa Cruz, CA), anti-PR antibody (Cat. No 8757 Cell Signaling Technology, Danvers, MA, USA), anti-MMP2 antibody (Cat. No 436000, Thermo Fisher Scientific, Waltham. MA, USA), and anti-MMP9 antibody (Cat. No.13667, Cell Signaling Technology, Danvers, MA, USA), anti-collagen IV antibody (Cat. No 14–9871-82, Thermo Fisher Scientific, Waltham. MA, USA). The secondary antibodies for immunohistochemistry were Mach2 anti-rabbit HRP (Biocare Medical, CA, USA), signal detection was achieved with diaminobencidin chromogen kit (Biocare Medical, CA, USA). The fluorochromes Alexa Fluor 488 donkey anti-mouse (Cat. No. A11029, Thermo Fisher Scientific, Waltham. MA, USA) or Alexa Fluor 594 goat anti rabbit (Cat. No. A11005, Invitrogen, Thermo Fisher Scientific, Waltham. MA, USA) were used for immunofluorescence.

The classification of the immunostaining of the tissue section was assessed by two independent observers in double blinded samples, detecting steroid hormone receptors in the nucleus of epithelial tumor cells and the presence of MMP-2 and MMP-9 in the epithelium and the stroma of the tumor. The immunoreactive score (IRS) of the sample is based on the percentage labeled cells (level 1, <10%; level 2,11–50%; level 3, 51–80%; and level 4, > 80%) and the staining intensity (+ to +++) within a range of 1 to12 [26], considering a sample as positive whenever it obtained an IRS ≥ 2.

### Statistical analysis

The frequency of MMPs in ovarian tumors was evaluated by comparison of proportions. The association between MMPs expression and hormone receptors steroid were analyzed in contingency tables using Chi-square and Fisher’s exact tests, as appropriate. The Kaplan-Meir analysis of survival time up to 6 years after diagnosis was performed in the whole population, evaluating significance by log rank test. Cox proportional hazard models were used for overall survival evaluation taking into consideration: age at diagnosis, histological subtypes, FIGO stages, type of surgery, AR, ERα, and PR expression, together with MMP-2 and MMP-9 in epithelium and stroma, analyzed as independent factors; additionally, multivariate models were designed with the significant variables. Statistical analyses were performed using SPSS statistics (v23, IBM, US) and Stata software (v14,Texas, US). A *P* value ≤0.05 was considered significant.

## Results

### Histological subtypes

**Fig. 1:**
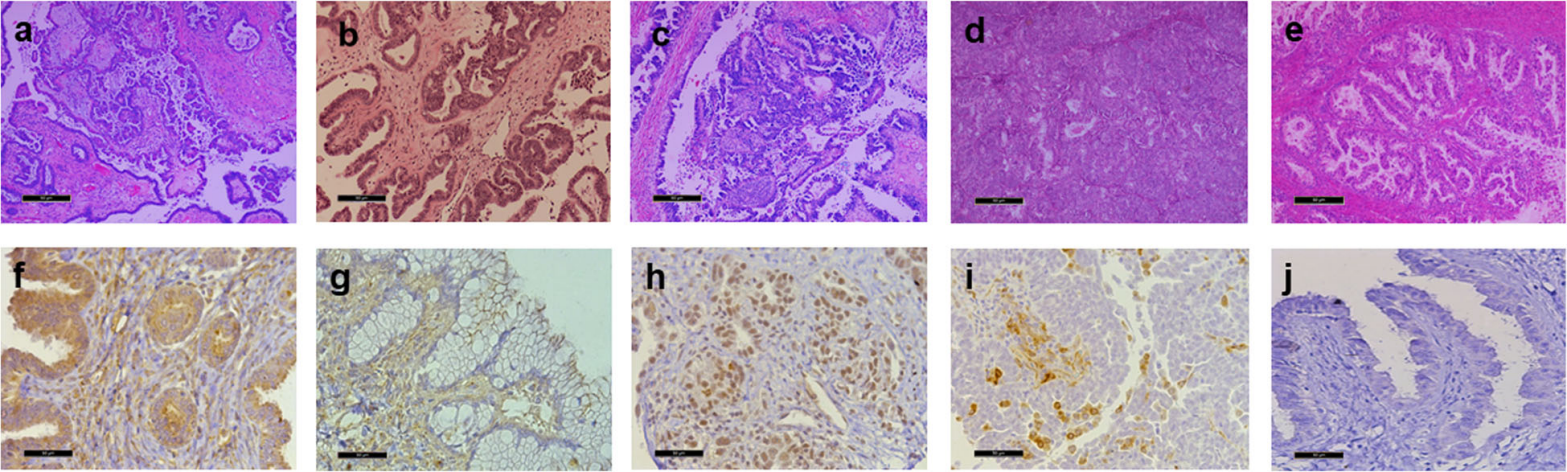
Representative hematoxilin-eosin stained sections. **a** Serous borderline tumor, **b** Low grade serous carcinoma, **c** High grade serous carcinoma, **d** Endometrioid carcinoma, and **e** Mucinous carcinoma. Immunohistochemistry for MMP-2 and MMP-9 in epithelial ovarian tumors. **f** MMP-2 in epithelium and stroma, of endometrioid tumor. **g** MMP-2 in stroma of mucinous tumor. **h** MMP-9 in epithelium of high grade serous carcinoma. **i** MMP-9 in stroma of endometrioid carcinoma. **j** negative control. Bars represents 50 μm

The following proportions of histological subtypes were observed in epithelial ovarian tumors: serous borderline tumor 20/88, high grade serous carcinoma 21/88, endometrioid carcinoma 21/88, mucinous carcinoma 11/88, and low grade serous carcinoma 9/88. Representative photomicrographs of each histological subtype and the immunohistochemistry for MMP-2 and MMP-9 are displayed in Fig. 1.

### Ovarian cancer cells express MMP-2 and MMP-9

**Fig. 2:**
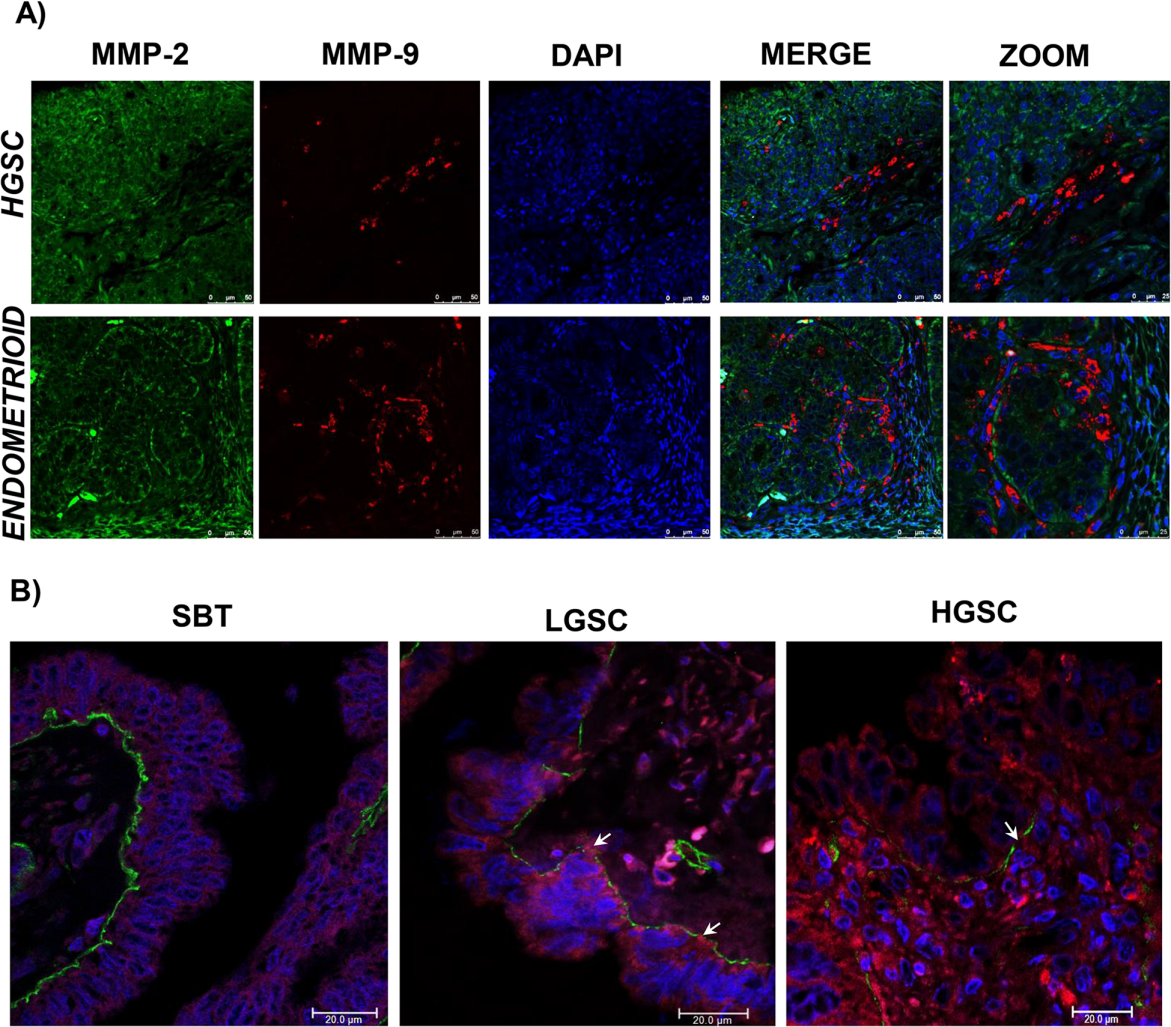
**a** Immunofluorescence for high grade serous carcinoma (HGSC) and endometrioid carcinoma. MMPs proteins were observed in the cytoplasm stained using MMP-2 antibody (green) and MMP-9 antibody (red), nuclei were stained with DAPI (blue). MMPs are shown in separate locations within the tumor. **b** Immunofluorescence for serous borderline tumor, low grade serous carcinoma (LGSC), and high grade serous carcinoma (HGSC). MMP-9 antibody (red) evidenced the cytoplasm of epithelial tumor cells, while the basal lamina was stained with collagen IV antibody (green) and the nuclei using DAPI (blue). Basal lamina discontinuities were observed in relation to MMP presence (arrows), mainly in LGSC

Differential frequency in the expression of MMP-2 and MMP-9 was observed in epithelial and stromal compartments considering the total of tumor samples: 85 and 56% in epithelium and 65 and 16% in stroma, respectively. The immunofluorescence for both metalloproteinases was observed in the epithelium and the stroma of the neoplasm with no co-localization being detected in high grade serous carcinoma and endometrioid carcinoma as shown in Fig. 2. Likewise,the presence of metalloproteinases in epithelial cells was associated to the degradation of the basal lamina (Fig. 2). Contingency table analysis of MMP-2 and MMP-9 frequency of expression in the total number of the tumor samples showed no association between both MMPs neither in the epithelium (*P* = 0.48) nor in the stroma of the tumor (*P* = 0.55).

### Expression of MMP-2 and MMP-9 in histological subtypes

**Fig. 3:**
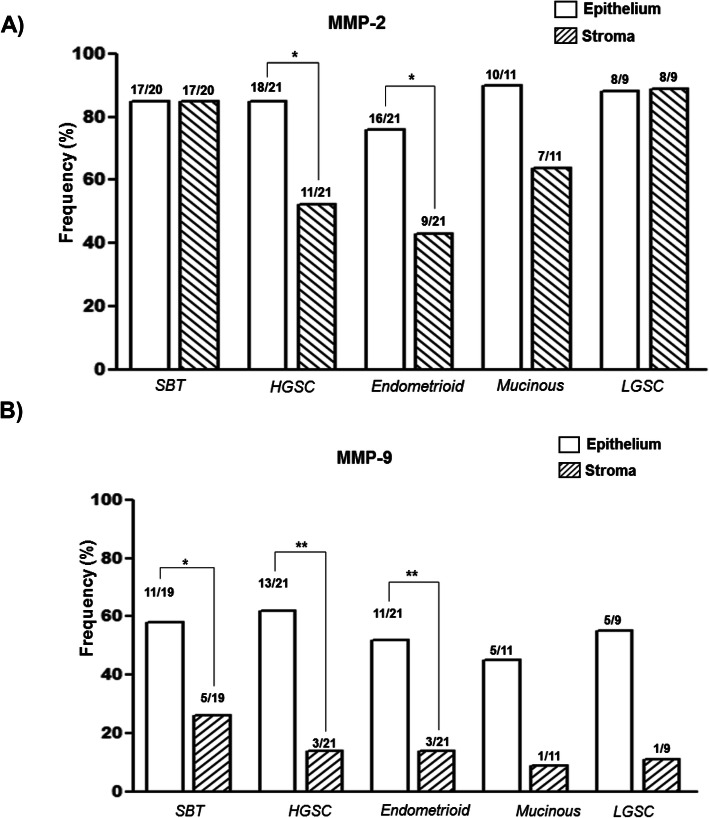
Frequency of expression of MMP-2 (**a**) and MMP-9 (**b**) in ovarian tumors. Bars represent the proportions of positive immunohistochemical reaction in epithelium and the stroma of tissue samples. Statistical analysis was performed using the test for contrasting the proportions of the groups,* *P* ≤ 0.05, ** *P* ≤ 0.01

The proportions of positive immunoreactions for MMP-2 and MMP-9 in ovarian tumor histological subtypes showed variations in the frequency of both metalloproteinases and, although no significant variations of MMP-2 were found in the epithelial location between histological subtypes, MMP-2 displayed a reduced frequency in the stroma compartments in high grade serous and endometrioid subtypes (Fig. 3). The frequency of MMP-9 was higher in epithelium compared to stroma, being significant in SBT, HGSC and endometrioid subtypes (Fig. 3) and a significant association between epithelium and stroma localization was observed for MMP-2 (*P* <0.001), however it was not observed for MMP-9 (*P* = 0.33).

### MMP-2 and MMP-9 association to sexual steroid receptors

The expression of MMP-2 and MMP-9 showed a significant association with the presence of androgen receptor in the epithelium of the total ovarian tumors. This relationship was not significant for estrogen receptor alpha or progesterone receptor (Fig. 1S). This same relationship was evaluated considering the histological subtypes resulting in a significant association between AR and MMP-2 in HGSC samples,13/13 AR positive tumors being also positive for MMP-2 (Table 2). Meanwhile, a significant association was observed between AR and MMP-9 in endometrioid carcinoma. A significant association between ERα and MMP-9 in LGSC was also registered and the presence of PR was positively associated with MMP-2 in serous borderline tumors. On the other hand, no significant association between MMP-2 and MMP-9 with steroid receptors was detected in mucinous carcinoma (Table 2). The association analysis of steroid hormone receptors with the frequency of MMP-2 and MMP-9 in the stroma of the histological subtypes of ovarian tumors showed no significant association in any combination, except for progesterone with MMP2 in SBT (Table 1S).

**Table 2:** Association between MMP protein expression and steroid hormone receptor in epithelium of the tumors by histological subtype

	*n*	AR			ERα			PR		
		*+*	*-*	*P*	*+*	*-*	*P*	*+*	*-*	*P*
**MMP-2**										
SBT	20	12/14	5/6	1.0	10/12	7/8	1.0	14/14	3/6	**0.018**
HGSC	21	13/13	5/8	**0.042**	5/6	13/15	1.0	10/11	8/10	0.586
Endometrioid	21	10/12	6/9	0.611	10/12	6/9	0.611	10/13	6/8	**1.0**
Mucinous	11	7/7	3/4	0.364	2/2	8/9	1.0	3/3	7/8	1.0
LGSC	9	7/7	1/2	0.222	4/5	4/4	1.0	6/7	2/2	1.0
**MMP-9**										
SBT	19	9/13	2/6	0.319	7/12	4/7	1.0	7/14	4/5	0.338
HGSC	21	8/13	5/8	1.0	4/6	9/15	1.0	7/11	6/10	1.0
Endometrioid	21	9/12	2/9	**0.030**	8/12	3/9	0.198	7/13	4/8	1.0
Mucinous	11	4/7	1/4	0.545	0/2	5/9	0.455	1/3	4/8	1.0
LGSC	9	4/7	1/2	1.0	1/5	4/4	**0.048**	1/2	4/7	1.0

*SBT* serous borderline tumor, *HGSC* high grade serous carcinoma, *LGSC* low grade serous carcinoma

*P* values obtained by Fisher’s exact test

### MMP-2 and MMP-9 association to FIGO tumor stage

The association of MMP-2 and MMP-9 located in epithelium and stroma, with the tumor stage according to FIGO, only demonstrated a significant association between the FIGO stage and MMP-2 located in the stroma of the tumor, with ovarian tumors classified at stages III and IV displaying a reduced presence of MMP-2 in the stroma of the tumor; all other comparisons were not significant (Table 3).

**Table 3:** Association between MMP-2 and MMP9 expression and the FIGO stage in ovarian tumor

			FIGO stages			*P*
	I	II	III	IV	Total	
**MMP-2**						
*Epithelium*	35/40	6/6	23/30	7/8	71/84	0.417
Stroma	31/40	6/6	15/30	4/8	56/84	**0.020**
**MMP-9**						
*Epithelium*	21/39	4/6	14/30	8/8	39/83	0.069
*Stroma*	8/39	0/6	4/30	2/8	14/83	0.526

Values obtained by Pearson Chi-Square

### Metalloproteinases and overall patient survival

The overall survival of the patients was analyzed using the Cox proportional hazard regression model, considering MMP-2 and MMP-9 in epithelium and stroma as predictive variables, together with the age of the patient, histological subtype, FIGO stage and steroid receptor expression of the tumors (Table 4). The obtained results showed that only MMP-2, age of the patient and FIGO stage were statistically significant risk factors for overall survival. Nevertheless, this was not the case for MMP-9, which was not significant as a predictive variable. Constructing multivariate models, MMP-2 and FIGO stages remained as significant variables. MMP-2 in epithelium resulted in a poor prognosis (HR: 11.49, CI _95%_ 1.42–92.7, *P* = 0.02) and MMP-2 in stroma displayed a protective effect (HR: 0.16, CI _95%_: 0.05–0.5, *P* = 0.002). FIGO stages are also an independent predictor of overall survival, (HR: 12.4, CI _95%_: 1.57–97.7, *P* = 0.02). The significance of HR for MMP-2 in epithelium and stroma was maintained in multivariate models that considered the age of the patient, histological subtype, and steroid receptor expression as covariates.

**Table 4:** Hazard ratio for metalloproteinases, sexual hormone receptors, and clinical characteristic in epithelial ovarian tumors

		Univariate			Multivariate	
Factor	HR	95% CI	*p*	HR	95% CI	*p*
MMP-2 epithelium	10.8	1.36–25.7	**0.02**	11.5	1.42–92.7	**0.02**
MMP-2 stroma	0.15	0.05–0.42	**<0.001**	0.16	0.05–0.50	**0.002**
MMP-9 epithelium	1.49	0.534.19	0.44	0.79	0.18–3.35	0.75
MMP-9 stroma	0.52	0.11–2.33	0.38	2.97	0.39–22.5	0.29
Androgen receptor	1.85	0.68–5.04	0.23	4.03	0.54–30.4	0.18
Estrogen receptor α	0.65	027–1.57	0.34	1.52	0.48–4.86	0.48
Progesterone receptor	0.95	0.40–2.37	0.95	1.41	0.36–5.82	0.61
FIGO stage	3.88	2.14–7.16	**<0.001**	12.4	1.57–97.7	**0.02**
Age at diagnosis	1.02	0.99–1.05	0.18	1.05	1.10–1.11	**0.03**
Histological subtype				3.15	0.73–13.7	0.13

*HR* Hazard ratio, *CI* confidence interval

**Fig. 4:**
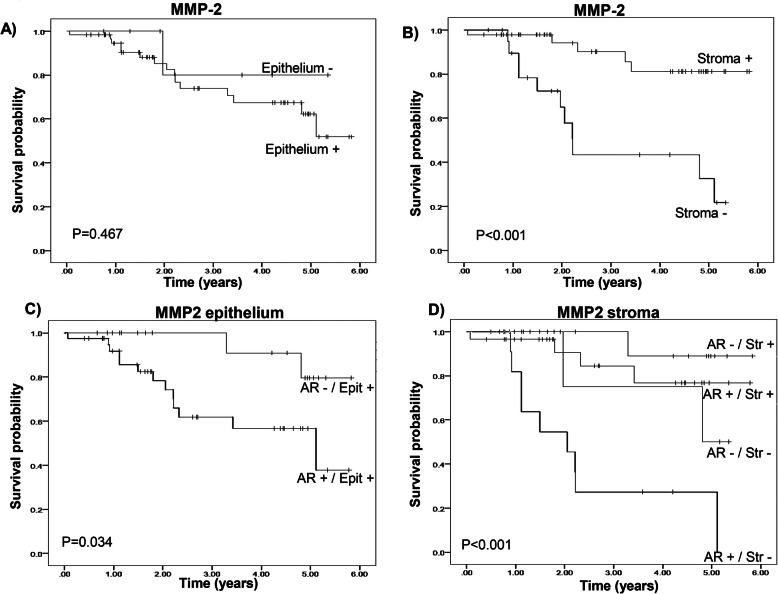
Kaplan-Meier survival analyses of patients with MMP-2 expression (pos./neg.) in epithelium (**a**) and stroma (**b**) and for the combination of AR expression with MMP-2 in epithelium (**c**) and in stroma (**d**) of the ovarian tumors. In epithelium, the combinations of AR expression (pos./ neg.) in tumors with positive MMP2 expression. In stroma, four categorical groups were analyzed for MMP-2 (pos./ neg.) with AR (pos./ neg.). Statistical significance is indicated in the figure

The survival rate observed in a Kaplan-Meier analysis of the patient with ovarian tumors, positive and negative for MMP-2 in epithelium and stroma of the tumor, corroborated a protective effect of the presence of MMP-2 in stroma, although no significant changes were observed regarding MMP-2 in epithelium (Fig. 4a, b).

Survival time of the combination of AR (pos/neg) with positive MMP-2 in epithelium, in Kaplan Meir analysis, displayed a significant reduction of survival in the combination AR (+) and MMP-2 (+) in epithelium (Fig. 4c) and the HR obtained through Cox regression analysis for the presence of AR(+) was (4.48 (CI_95%_: 0.99–1.99, *P* = 0.051). The combination of MMP-2 in stroma (pos/neg) with AR (pos/neg) resulted in a significant reduction of survival time in the AR (+) and MMP-2 (−) tumors (Fig. 4d). Cox regression analysis of the four combination levels resulted in only significant for AR (+) and MMP-2 (−) in stroma (HR: 5.85 CI_95%_: 1.89–18.1, *P* = 0.002).

## Discussion

The analysis of this cohort provides evidence of the presence of MMP-2 and MMP-9 in the epithelium and stroma of ovarian tumors, showing variations in the presence of MMPs when stratified by histological subtypes, FIGO stages and expression of AR, ERα, and PR in the ovarian tumor as well.

Immunofluorescence for MMP-2 and MMP-9 observed using confocal microscopy indicates the absence of co-localization between both endopeptidases at least in HGSC and endometrioid carcinoma, an observation confirmed by the lack of association between the presence of both metalloproteinases in the ovarian tumors. A differential intracellular location of both metalloproteinases has been previously described [27] in which variation in production, storage and secretion mechanism; could explain changes in the location of MMP-2 and MMP-9 [28].

The association of MMP-2 and MMP-9 with the expression of AR in the ovarian tumor analyzing the whole population is interesting as it provides evidence of the possible significance of the androgen receptor in ovarian tumors, being the most frequent receptor in ovarian cancer [20] associated to MMP-2 and MMP-9 which are related to the invasion and aggressiveness of the tumor [28]. Therefore, the reduction of survival time and the increase of HR in the combination of positivity of AR and MMP-2 in epithelium in the tumors could be related to this interaction. Although further information will be required in order to explain the relationship between AR and MMP-2 in ovarian tumors.

Interestingly, in the analysis by histological subtypes, AR is mainly associated to MMP-2 in HGSC and significantly associated to MMP-9 in endometrioid carcinoma. Moreover, MMP-2 is associated to PR in SBT while ERα is linked to MMP-9 in LGSC. These variations between histological subtypes confirm the postulation that epithelial ovarian tumor subtypes are particular diseases with the same anatomical location [3, 29].

Furthermore, the results displaying the presence of MMP-9 which demonstrated the absence of co-localization with MMP-2, the particular association with steroid receptors in histological subtypes, the lack of association of MMP-9 with FIGO stages and the lack of significance for MMP-9 in Cox proportional hazard analysis indicate that MMP-2 and MMP-9, although having common function as “gelatinases”, differ in their significance in epithelial ovarian tumors and probably show variations in the interaction with sexual steroid receptors. Previous studies demonstrating tissue specificity and variation in the regulation and functions of MMP-2 and MMP-9 [5, 28] could be the explanation of the present findings.

The presence of MMP-2 in the epithelium and stroma of the tumor has a particular distribution in the subtypes of ovarian tumors; in the case of LGSC and the borderline serous tumor the presence of MMP-2 in stroma is similar to the epithelium. On the contrary, in HGSC and endometrioid carcinoma the frequency of MMP-2 in stroma is almost reduced by half, probably in relation to the malignancy of both subtypes, although this particular distribution is not observed for MMP-9. Interestingly, the frequency of MMP-2 presence is significantly reduced in the stroma of ovarian tumors classified as FIGO stages III and IV. These findings, along with the results provided by Cox hazard proportional regression support the importance of MMP-2 as a prognostic biomarker for epithelial ovarian tumor, where MMP-2 in the stroma is an independent predictor of a reduced risk of death. On the other hand, MMP-2 in the epithelial cells of the ovarian tumor predicts an increased risk of death. This is not the case for MMP-9 that does not have any significance in univariate or multiTvariate analyses with Cox regression. In literature there are controversial results on the HR of MMP-2 and MMP-9 [14, 17, 18], but ours agree with meta-analysis concluding that MMP-2 has a prognostic value in ovarian cancer [17].

Kaplan-Meier analysis shows that the overall survival time of the patients with ovarian tumors is increased by the presence of MMP-2 in the stroma, which has a protective effect indeed. The survival function estimation after 5 years is 0.81 in positive stroma and 0.33 in the negative one, results which are closely similar to those reported previously in Finnish population [13]. The protective effect observed with the presence of MMP-2 in the stroma of the tumor could be explained by the secretion of anti-angiogenic factors [30], the production of chemoattractant for immuno-competent cells [31] or the increase in the secretion of TGFβ [32], all of which have been described to be associated with MMP-2. On the other hand, the production of MMP-2 in the epithelial tumor cells is involved in basal lamina degradation and facilitation of ovarian cell adhesion to both, peritoneun and omentum [33, 34].

The importance of AR in ovarian cancer has been previously explored [35]. In the present cohort, the expression of AR per se in the tumor cells did not modify patient survival. However, the presence of AR in the positive MMP-2 in tumor epithelium reduced survival in patients. Moreover, AR expression reduces patient survival whenever the protective effect of MMP-2 in the tumor stroma was absent; therefore, a deleterious effect mediated by AR associated to MMPs should be considered in ovarian cancer studies.

## Conclusions

Based on the present results we conclude that MMP-2 located in the epithelium and the stroma are independent prognostic biomarkers for overall survival in epithelial ovarian tumors. The presence of MMP-2 in the stroma of the tumor is a protective factor while the presence of MMP-2 in the epithelium indicates an unfavorable prognosis. The presence of AR together with MMP-2 in the tumor cells is a risk factor to be considered in epithelial ovarian tumors.

## Supplementary information

**Additional file 1: Table S1.** Association between MMPs proteins expression and steroid hormone receptors in stromaof the tumors by histological subtype.

**Additional file 2: Figure S1.** MMP-2 and MMP-9 association to AR, ER alpha and PR

## Data Availability

The data set used and/or analyzed during the current study are available from the corresponding author on reasonable request

## Abbreviations

MMP: Matrix metalloproteinase
LGSC: Low grade serous carcinoma
HGSC: High grade serous carcinoma
SBT: Serous borderline tumor
ERα: Estrogen receptor alpha
PR: Progesterone receptor
AR: Androgen receptor
IRS: Immunoreactive score
