# Supplementary material for: Expression of metalloproteinases MMP-2 and MMP-9 is associated to the presence of androgen receptor in epithelial ovarian tumors
Source: J Ovarian Res. 2020 Jul 28;13:86. doi: 10.1186/s13048-020-00676-x (PMC7385964; doi:10.1186/s13048-020-00676-x)
Supplement: Supplementary file 1 — Additional file 1: Table S1. Association between MMPs proteins expression and steroid hormone receptors in stromaof the tumors by histological subtype. [file 13048_2020_676_MOESM1_ESM.docx]

Table 1S. Association between MMPs proteins expression and steroid hormone receptors in stromaof the tumors

by histological subtype.


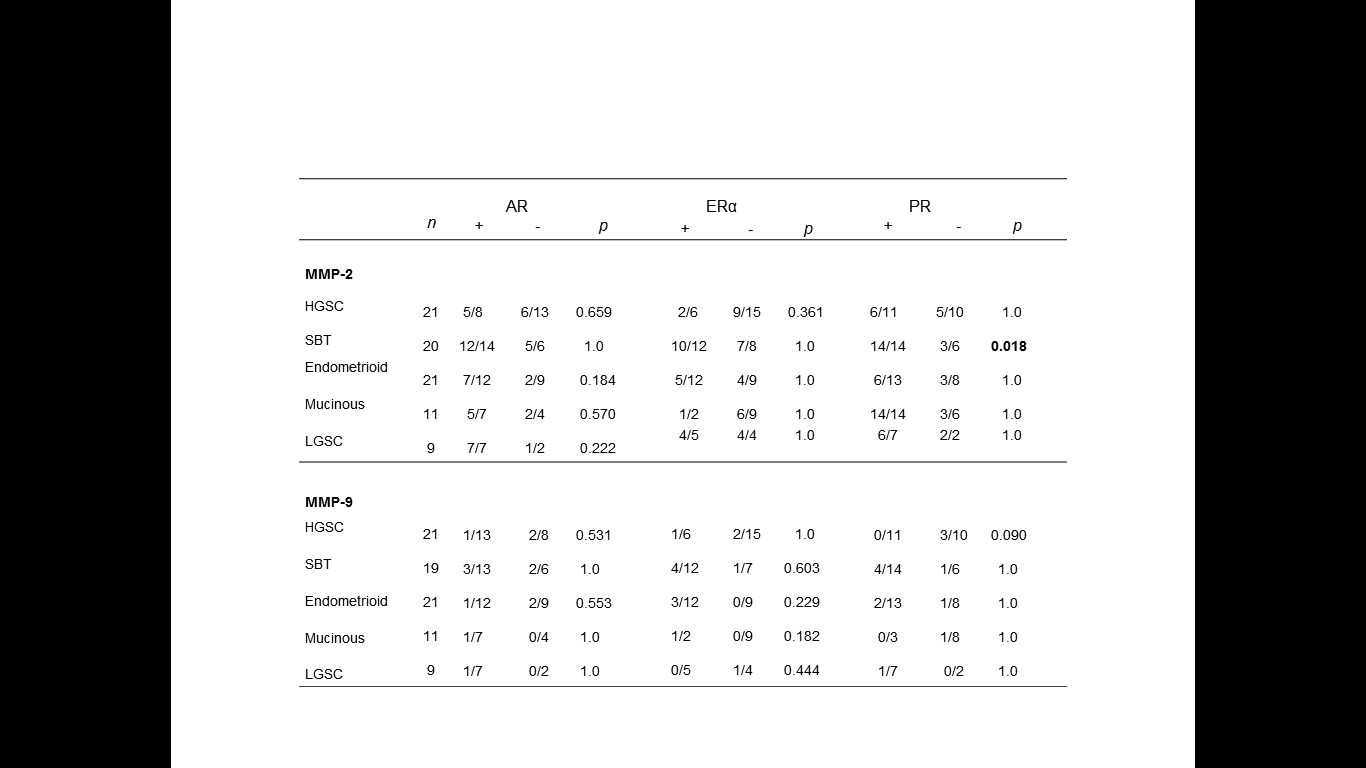


SBT: serous borderline tumor, HGSC: high grade serous carcinoma , LGSC: low grade serous carcinoma

P values obtained by Fisher’s exact test
