# Supplementary figures and images for: Expression of metalloproteinases MMP-2 and MMP-9 is associated to the presence of androgen receptor in epithelial ovarian tumors
Source: J Ovarian Res. 2020 Jul 28;13:86. doi: 10.1186/s13048-020-00676-x (PMC7385964; doi:10.1186/s13048-020-00676-x)

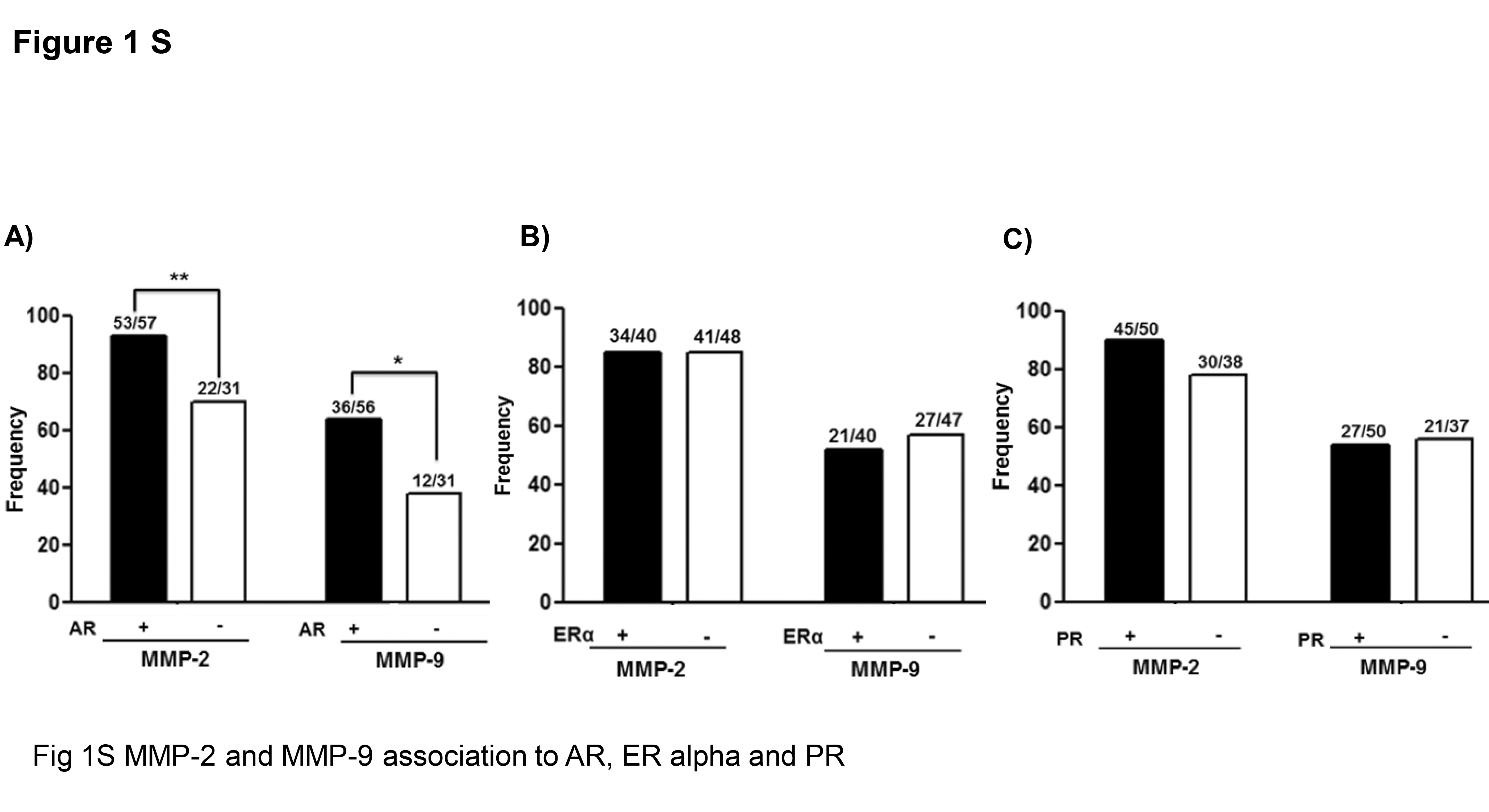

Supplement: Supplementary file 2 — Additional file 2: Figure S1. MMP-2 and MMP-9 association to AR, ER alpha and PR [file 13048_2020_676_MOESM2_ESM.tif]
